# Tackling the challenges of matching biomedical ontologies

**DOI:** 10.1186/s13326-017-0170-9

**Published:** 2018-01-15

**Authors:** Daniel Faria, Catia Pesquita, Isabela Mott, Catarina Martins, Francisco M. Couto, Isabel F. Cruz

**Affiliations:** 1 Instituto Gulbenkian de Ciência, R Quinta Grande 6, Oeiras, Portugal; 2 LASIGE, Faculdade de Ciências, Universidade de Lisboa, Campo Grande, Lisboa, Portugal; 3 School of Computer Science, University of Manchester, Oxford Rd, Manchester, UK; 4 ADVIS Lab, Department of Computer Science, University of Illinois at Chicago, 851 S. Morgan St., Chicago, Illinois USA

**Keywords:** Ontology matching, Biomedical ontologies

## Abstract

**Background:**

Biomedical ontologies pose several challenges to ontology matching due both to the complexity of the biomedical domain and to the characteristics of the ontologies themselves. The biomedical tracks in the Ontology Matching Evaluation Initiative (OAEI) have spurred the development of matching systems able to tackle these challenges, and benchmarked their general performance. In this study, we dissect the strategies employed by matching systems to tackle the challenges of matching biomedical ontologies and gauge the impact of the challenges themselves on matching performance, using the AgreementMakerLight (AML) system as the platform for this study.

**Results:**

We demonstrate that the linear complexity of the hash-based searching strategy implemented by most state-of-the-art ontology matching systems is essential for matching large biomedical ontologies efficiently. We show that accounting for all lexical annotations (e.g., labels and synonyms) in biomedical ontologies leads to a substantial improvement in F-measure over using only the primary name, and that accounting for the reliability of different types of annotations generally also leads to a marked improvement. Finally, we show that cross-references are a reliable source of information and that, when using biomedical ontologies as background knowledge, it is generally more reliable to use them as mediators than to perform lexical expansion.

**Conclusions:**

We anticipate that translating traditional matching algorithms to the hash-based searching paradigm will be a critical direction for the future development of the field. Improving the evaluation carried out in the biomedical tracks of the OAEI will also be important, as without proper reference alignments there is only so much that can be ascertained about matching systems or strategies. Nevertheless, it is clear that, to tackle the various challenges posed by biomedical ontologies, ontology matching systems must be able to efficiently combine multiple strategies into a mature matching approach.

**Electronic supplementary material:**

The online version of this article (doi:10.1186/s13326-017-0170-9) contains supplementary material, which is available to authorized users.

## Background

The biomedical domain presents a strong case for the application of ontology matching, as there are hundreds of biomedical ontologies which were mostly developed independently, and many of them cover overlapping domains [1]. Establishing meaningful links between such ontologies is critical to ensure interoperability and has the potential to unlock biomedical knowledge by bridging siloed data. However, biomedical ontologies present some of the most significant challenges to the field of ontology matching, given their characteristics and the complexity of the domain they cover.

The first hurdle that ontology matching systems must overcome to match biomedical ontologies is their large size. Many of the most widely used biomedical ontologies have tens of thousands of classes (e.g., the Gene Ontology, the Uber Anatomy Ontology) or even hundreds of thousands (e.g., the SNOMED Clinical Terms, the Chemical Entities of Biological Interest Ontology). Handling such large ontologies presents computational challenges throughout the ontology matching pipeline. Matching systems must first be able to load the ontologies in a memory efficient manner, then circumvent the quadratic complexity of the matching problem, and finally be able to effectively select the final set of mappings from a potentially large universe of plausible mapping candidates. Without tackling these challenges, ontology matching systems cannot match large biomedical ontologies in practice.

Another challenge in matching biomedical ontologies is the rich and complex vocabulary of the biomedical domain. Biomedical ontologies possess a rich lexical component, with each class being typically described by several annotations such as labels and different types of synonyms (e.g., exact, broad, narrow, related). For example, the Uber Anatomy Ontology (UBERON) class UBERON_0000948 has amongst its annotations: label “heart”, exact synonyms “vertebrate heart” and “chambered heart”, narrow synonym “branchial heart”, related synonym “cardium”, and even relational adjective “cardiac” [2]. Taking into account this lexical complexity is critical for matching biomedical ontologies effectively. On the one hand, matching systems must make use of all these annotations to obtain a reasonable recall, as what is a label in one ontology may be a synonym in another. On the other hand, systems must be able to account for the different specificity of the various types of annotations to successfully navigate through the many cases of homonymy, paronymy, and overlapping words, and thus attain a high precision. For example, the Foundational Model of Anatomy (FMA) class 59762 has label “gingiva” and exact synonym “gum” [3], but the word gum also has different meanings in the biomedical context. It can refer to a dietary gum, as is the case of the National Cancer Institute Thesaurus (NCI) class C68500 which has “gum” as exact synonym [4], and also to a type of drug preparation, as is the case of the SNOMED Clinical Terms (SNOMED) class 426210003, which has “gum” as a label [5]. However, the last two ontologies also have a class with label “gingiva” (and synonym “gum”), so this case illustrates how valuing label-to-label mappings over mappings involving synonyms would enable matching systems to find the correct mappings and avoid the incorrect ones when matching either of these two ontologies with FMA.

It is common across all domains that different ontologies have different modeling views of a given domain. However, the complexity of the biomedical domain makes this particularly challenging. Biomedical ontologies on the same domain can have profound differences in organization to the point that they are logically irreconcilable due to conflicting restrictions. For instance, in NCI, anatomical structures and proteins are modeled as disjoint, and consequently the fibrillar or filamentous form of actin (class C32581) which is an anatomical structure is disjoint with the actin protein (class C16258). By contrast, in FMA, proteins are modeled as anatomical structures, and fibrillar actin (class 67844) is actually a subclass of the actin protein (class 67843). Thus, while it would be biologically correct to map the classes describing the fibrillar form of actin and the classes describing the actin protein of each ontology, doing so would cause logical conflicts if the two ontologies were integrated in this way. This example illustrates the trade-off between completeness and logical soundness that often must be considered when matching biomedical ontologies [6].

The simple but particular semantics of biomedical ontologies is another aspect that differentiates them from the ontologies of other domains. Most biomedical ontologies have few properties and relatively simple semantics – for instance, half of the ontologies in BioPortal fit into the tractable OWL2EL profile [7]. This fact, together with the lexical richness and frequent modeling differences of biomedical ontologies, means that strategies for matching these ontologies tend to rely primarily on lexical matching algorithms, with structural matching algorithms taking a secondary role, if employed at all. However, when they are employed, structural matching algorithms should take into consideration an object property of particular importance in biomedical ontologies—“part of”— which often accounts for a second hierarchical backbone (a partonomy) in complement of the taxonomic backbone defined by subclass relations.

While the specialized biomedical vocabulary may render general purpose lexical tools such as WordNet ineffective, the increasing profusion of biomedical ontologies means that there are usually abundant sources of background knowledge available to ontology matching systems in the form of external related ontologies. The challenge lies in identifying the most suitable and useful sources of background knowledge among potentially hundreds of candidate ontologies. Addressing this challenge has been the topic of several studies which proposed metrics for estimating the usefulness of background ontologies [8, 9]. Of particular relevance as sources of background knowledge are the efforts of the OBO Foundry to include external references in their ontologies [10]. There are two forms of these: direct cross-references to other ontologies, and logical definitions that correspond to composite references to two or more other ontologies. Both are manually curated, high-quality knowledge sources that can be reused as background knowledge by ontology matching systems.

The relevance of the biomedical domain for ontology matching and the interesting challenges it raises have motivated the inclusion of a growing number of biomedical ontology alignment tasks in the Ontology Matching Evaluation Initiative (OAEI). These tracks have played a key role in driving forward the development of systems and strategies to tackle many of the challenges of matching biomedical ontologies. While the OAEI has done an excellent job at evaluating ontology matching at the system level, assessing the contributions of the various strategies implemented by each system is beyond its scope, as each system is different and the level to which systems can be broken down into individual strategies varies.

In the interest of providing a more in-depth evaluation, in this study we dissect the strategies employed by matching systems to tackle the aforementioned challenges of matching biomedical ontologies and gauge the impact of the challenges themselves on matching performance. We use AgreementMakerLight (AML) [11] as a platform for the study, as it meets three critical criteria: it is one of the top performing systems in the biomedical tracks of the OAEI [12] and thus represents the state of the art; it was designed specifically for matching biomedical ontologies and thus to tackle most of the challenges involved therein; and it has a modular architecture, which is essential to enable the type of analysis we aim to conduct in this study. It is also the matching system with which we are most familiar, thus facilitating our work.

The rest of the manuscript is organized as follows: in the “Related work” section we review how matching systems participating in the OAEI have tackled the challenges of matching biomedical ontologies; in the Methods, we provide a brief overview of AML, make an in-depth analysis of the strategies AML and other top-performing matching systems employ to tackle biomedical ontologies, and describe the datasets and experimental setting; in the Results and Discussion we dissect the impact of several of the strategies implemented by AML on its effectiveness and efficiency; and finally, in the Conclusions, we provide an overarching view of the study and ponder on the aspects where the state of the art in matching biomedical ontologies can be improved.

### Related work

Throughout the history of the OAEI, a number of biomedical ontology alignment tasks have been introduced and multiple matching systems have participated in them. The *Anatomy* track was introduced in the first OAEI proper, in 2005. The *Large Biomedical Ontologies* track was introduced in OAEI 2011.5 with two tasks, and expanded to six tasks in OAEI 2012. More recently, the *Disease and Phenotype* track was introduced in 2016 with two tasks, and expanded to four tasks in 2017. Of the many systems that have participated in one or more editions of one of these tracks, 27 have a peer-reviewed publication and thus can be reviewed with respect to how they address the challenges outlined in the previous section. Table 1 summarizes this information.

**Table 1:** Overview of the ontology matching systems that participated in OAEI biomedical tracks

System	Size	Lexicon	Relations	Repair	Background knowledge	OAEI Bio tracks
AgrMaker [19]	+	weights	*part of*	-	Bio; Man; Med	A
AML [11]	+++	WN; weights	all	Logic	Bio; Auto; M/E	all
Anchor-Flood [40]	+	WN	-	-	Man; Exp	A
Aroma [41]	+++	-	-	-	-	A, LB
ASMOV [42]	+	WN	-	-	Bio; Man; Exp	A
AUTOMSv2[43]	++	WN; weights	-	-	Man; Exp	LB-
BLOOMS [20]	+	WN	*part of*	-	Bio; Man; Med	A
COMMAND [44]	+	-	-	Logic	-	A
CroMatcher [45]	+	WN	-	-	Man; Exp	A
DKP-AOM [46]	+	WN	-	-	Man; Exp	A, LB-
DSSim [47]	+	WN	-	-	Man; Exp	A
FCA-Map [16]	++	UMLS	-	Logic	Man; Exp	all-
GMap [48]	+	external?	-	Logic	Man; Exp	A
GOMMA [49]	+++	-	-	-	Bio; Auto; Med	A, LB
kosimap [50]	+	-	-	-	-	A
LogMap [13]	+++	WN; UMLS	-	Logic	Bio; Auto; M/E	all
LP HOM [51]	+	-	-	-		A
Lyam++[52]	++	BabelNet	-	-	Man; Exp	all-
MapPSO [53]	+	-	-	-	-	A
OACAS [54]	+	-	-	-	-	A
PhenomeNET [21]	++	(AML)	*part of*	-	Bio; Man; Med	DP
SAMBO [22]	+	WN	*part of*	-	Bio; Man; Exp	A
ServOMap [55]	+++	WN	-	Logic	Man; Exp	A, LB
TaxoMap [56]	+	-	-	-	-	A
TOAST [57]	+	-	-	-	-	A
WikiMatch [58]	++	Wikipedia	-	-	Man; Exp	A, LB-
YAM++[59]	+++	WN	-	Rules	Man; Exp	A, LB

*Size* details the systems’ capability of handling medium-sized (+), large (++) or very large (+++) ontologies; *Lexicon* lists their use of lexical tools such as WordNet (WN) or the UMLS SPECIALIST Lexicon (UMLS), as well as whether they use synonym weights; *Relations* lists the types of relations they contemplate in addition to subclass relations; *Repair* details whether they perform alignment repair based on logic or rules; *Background Knowledge* describes whether they use biomedical ontologies as background knowledge (Bio), whether the process of background knowledge selection is manual (Man) or automatic (Auto), and whether background knowledge is used as a mediator (Med) or for lexical expansion (Exp); *OAEI Bio Tracks* lists the tracks in which the system successfully competed in out of Anatomy (A), Large Biomedical Ontologies (LB), and Disease & Phenotype (DP), with - indicating that the system did not complete the largest LB tasks

Handling the largest biomedical ontologies continues to prove a tough challenge for ontology matching systems. Of the surveyed systems, only 5 have been able to complete the largest tasks of the *Large Biomedical Ontologies* track at some point in their history. In 2016, only AML and LogMap [13] did so out of 8 independent participants [14]. Although this isn’t often detailed in publication, most systems that are able to tackle large ontologies make use of data structures with inverted indices that enable hash-based searching rather than pairwise matching, and thus circumvent the quadratic nature of ontology matching.

The lexical complexity of biomedical ontologies is also an aspect which few ontology matching systems are prepared to tackle. Most systems do make use of the WordNet [15], but this is a general purpose English lexical tool, so while it may enable systems to find some lexical variants, its coverage of the specialized biomedical vocabulary is far from comprehensive. Thus, a few matching systems such as FCA-Map [16] and LogMap [13] opt for the domain specific UMLS SPECIALIST Lexicon [17]. The diversity of ontology annotations contemplated by each matching system is unclear, but the use of weights for synonyms is an uncommon feature, and a differentiating weighting scheme to reflect the precision of different types of lexical annotations has only been reported in AML [18].

Only a few systems consider “part of” relations when employing structural matching algorithms: AgreementMaker [19], BLOOMS [20], PhenomeNET [21] and SAMBO [22] consider it explicitly, whereas AML considers all relations.

The relevance of alignment coherence, which is to say, an alignment that when used to integrate the input ontologies does not lead to logical unsatisfiabilities, has been gaining traction within the ontology matching community. The number of matching systems that either implement or reuse an alignment repair algorithm, while still relatively low, has increased in recent years. Unfortunately, the OAEI has not been able to provide a testing ground for alignment repair that highlights the conflict between completeness and logical coherence [23], as manually curated reference alignments are not available for the tasks in which alignment repair is a meaningful problem. Until such a test is created, the evaluation of alignment repair algorithms will remain superficial, and their use will remain almost exclusively automated.

While the use of background knowledge is very common among ontology matching systems, we include as background knowledge the usage of WordNet or the SPECIALIST lexicon for lexical expansion (i.e., to enrich the input ontologies with synonyms). The use of biomedical ontologies (counting the UMLS Metathesaurus [17]) as background knowledge sources is less common, occurring in only 8 of the surveyed systems. Most systems that use background knowledge employ fixed manually selected sources, with only AML, GOMMA [9] and the LogMapBio variant [24] implementing an automatic selection algorithm. The latter deserves particular mention in that it makes use of BioPortal’s [1] search engine and thus has access to virtually any biomedical ontology as background knowledge. The majority of the systems that use background knowledge make use of it for lexical expansion, as that is the main usage of the WordNet. Of the systems that employ biomedical ontologies as background knowledge, most use these ontologies as mediators, by mapping the background ontology to the background knowledge ontology, and then intersecting the two background alignments to generate an alignment between the input ontologies.

## Methods

### AML overview

AML is an ontology matching system originally developed to tackle the challenges of matching large biomedical ontologies [11], as its namesake and predecessor AgreementMaker [19] was not designed to handle ontologies of this size. While AML’s scope has since expanded, biomedical ontologies have remained one of the main drives behind its continued development.

**Fig. 1:**
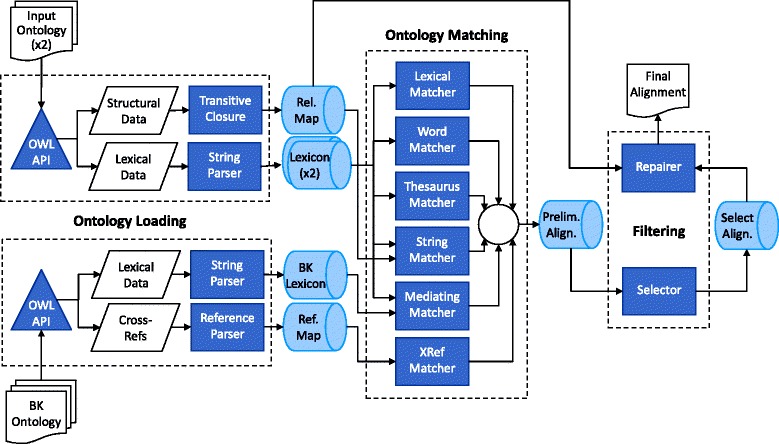
Flowchart representation of AML’s pipeline. The pipeline is divided into three stages: ontology loading, where input or background knowledge (BK) ontologies are parsed and loaded into AML’s data structures; ontology matching, where matching algorithms generate mapping candidates which are combined into a preliminary alignment; and filtering, where problem-causing mappings are removed from the preliminary alignment to produce a final alignment

AML’s ontology matching pipeline is divided into three phases: ontology loading, matching, and filtering. The pipeline is illustrated in Fig. 1.

In the ontology loading phase, the input ontologies are loaded using the OWL API [25], then parsed into AML’s data structures [11]. The most important of these are the *Lexicon*, which stores all the lexical information of an ontology in normalized form, and the *RelationshipMap*, which stores the structural information.

In the matching phase, AML’s various matching algorithms (or matchers) are executed and combined. These include [11, 12, 26]:

- The *LexicalMatcher*, which finds literal full-name matches between the *Lexicon* entries of two ontologies.
- The *WordMatcher*, which finds matches between entities by computing the word overlap between their *Lexicon* entries.
- The *StringMatcher*, which finds matches between entities by computing the string similarity between their *Lexicon* entries using the ISub metric [27].
- The *ThesaurusMatcher*, which find literal full-name matches involving synonyms inferred from an automatically generated thesaurus, as we will detail in the next subsection.
- The *MediatingMatcher*, which employs the *LexicalMatcher* to align each of the input ontologies to a third background ontology, and then intersects those alignments to derive an alignment between the input ontologies.
- The *XRefMatcher*, which is analogous to the *MediatingMatcher*, but relies primarily on OBO [10] cross-references between the background ontology and the input ontologies.
- The *LogicalDefMatcher*, which matches classes that have equal or corresponding OBO [10] logical definitions, as we will detail in a subsequent subsection.

In the filtering phase, AML applies algorithms that remove problem-causing mapping candidates from the preliminary alignment to generate the final alignment. The problems that are addressed include cardinality conflicts (i.e., cases where a class of one ontology is mapped to more than one class of the other ontology) and logical conflicts (i.e., cases where two or more mappings cause the input ontologies to become unsatisfiable when merged via those mappings).

Cardinality conflicts are resolved using the heuristic *Selector* algorithm, which selects mappings in descending order of similarity score in one of its three modes: ‘strict’, in which all cardinality conflicts are resolved; ‘permissive’, which accepts cardinality conflicts in the case of similarity score ties; and ‘hybrid’, which accepts conflicting pairs of mappings with high similarity score (above 0.75) and otherwise behaves as the ‘permissive’ mode [11]. Logical conflicts are resolved by the *Repairer* algorithm [23].

### Handling large ontologies

There are three key strategies implemented by AML and other efficient ontology matching systems to match large ontologies: hash-based searching, parallelization, and search space reduction. Additionally, large ontologies also pose problems with respect to the memory requirements of the similarity matrix.

#### Hash-based searching

The hash-based searching strategy is the most critical strategy for scalability, as it effectively reduces the time complexity of the matching problem from quadratic to linear. This strategy relies on using data structures based on *HashMaps*, with inverted indices, to store the lexical information of the ontologies. By inverted indices, we mean that rather than having the class ids as keys, their lexical attributes (e.g., the various labels and synonyms, or the words these contain) are used as keys and the values are the sets of ids of the classes that have each attribute. This enables matching systems to simply check whether each lexical attribute of one ontology occurs in the other, rather than making pairwise comparisons of the classes of the two ontologies. Since the attributes are *HashMap* keys, and *HashMap* access normally has *O*(1) time complexity, the hash-based searching strategy has *O*(*n*) complexity overall, where *n* is the number of lexical attributes in the ontology with the least attributes. By contrast, the traditional pairwise matching strategy has *O*(*mn*) complexity where *m* and *n* are the number of lexical attributes in the two ontologies to match.

The one limitation of hash-based searching is that it is usually restricted to finding equal attributes—at least when default Java *String* hash keys are used, as is the case in AML. Thus, it can be employed for literal full-name matches (*LexicalMatcher*), for matches based on overlapping words (*WordMatcher*), or even overlapping n-grams (not implemented in AML), but not for traditional string similarity comparisons (*StringMatcher*). Moreover, the effectiveness of the hash-based searching strategy hinges heavily on normalizing the lexical attributes a priori, in order to maximize the number of equal entries found.

In the case of AML, lexical attributes are normalized upon entry in the *Lexicon*, during the ontology loading stage. This normalization consists in removing all non-word non-digit characters (except parentheses and dash), inserting white spaces where capitalization is found within words (e.g., “hasPart” becomes “has Part”), and finally converting all characters to lower case. However, because biomedical ontologies may include special formulas (chemical or otherwise), AML uses patterns to detect whether a lexical attribute is a normal word-based name or a formula. In the latter case, the only normalization done is the replacement of underscores with white spaces.

#### Parallelization

Parallelization is a common strategy for improving computational efficiency that exploits the multi-core architecture of modern CPUs. In the context of ontology matching, it typically consists on distributing the computational load by the available cores by either running different (matching) algorithms in parallel or dividing an algorithm into a set of tasks and running those in parallel. While parallelization does not affect the computational complexity of the underlying algorithms, it can reduce their execution time by a factor of up to N, where N is the number of available CPU cores.

AML’s *StringMatcher* and *Repairer* algorithms are both implemented for parallelization via subdivision into parallel tasks, given that they are the two main bottlenecks in AML’s matching pipeline. AML’s remaining matching and filtering algorithms are not implemented for parallelization because they have linear complexity and run in at most a few seconds for even the largest ontologies, so the gain in parallelizing them would be negligible to AML’s total run time.

#### Search space reduction

Under search space reduction, we include the two families of strategies that aim to reduce the search space of the ontology matching problem—partitioning and pruning—as well as the strategy that aims to reduce the scale of the alignment repair problem—modularization.

Partitioning or blocking consists in dividing the ontologies into (usually vertical) partitions or blocks in order to transform a single large matching problem into several smaller ones [28]. Its simplest application is to reduce the memory requirements of the matching task, as is the case in AML’s *WordMatcher* algorithm. However, it can also be used to reduce the search space of the matching problem by determining which blocks have a significant overlap (typically using a hash-based searching strategy) and attempting to match only those [29]. In this application, it can improve not only the efficiency but also the effectiveness of the matching process, by excluding false positives.

Pruning encompasses any strategy that dynamically avoids comparing parts of the ontologies without partitioning them beforehand [28]. The most common of these strategies is precisely hash-based searching, as it effectively only makes comparisons between entities that have equal *HashMap* indices (be they names, words, or n-grams). In addition to this form of pruning, AML employs another form called local matching when applying traditional pairwise matching algorithms (such as the *StringMatcher*) to large ontologies. This strategy consists of matching entities only in the neighborhood of mapped entities found using more efficient (and reliable) hash-based search algorithms. Like blocking, it not only improves computational efficiency but can also help filter false positives.

Modularization consists of identifying the classes that are semantically relevant for determining whether an alignment is coherent in order to reduce the search space of the repair problem. It is akin to partitioning, but is carried out after the matching stage, and contemplates both the input ontologies and the alignment between them. To enable modularization and reduce the complexity of the repair problem, repair algorithms tend to consider simplifications of the Description Logic of OWL—for instance, the repair algorithms of both AML and LogMap are based on propositional logic [13, 23]. AML’s modularization reduces the search space of the repair problem both with regard to the classes that must be tested for satisfiability (since most tests are logically redundant) and with regard to the classes that must be searched (only those with multiple parents, or involved in mappings or logical restrictions) [23].

#### Similarity matrices

Another consideration that is critical for matching large ontologies is that the memory requirement of a similarity matrix between two ontologies scales quadratically with their size. For example, for the *FMA-SNOMED whole* task of the OAEI large biomedical ontologies track, the similarity matrix would require an unwieldy 72 GB RAM if similarity scores were stored with 8 Byte precision. The strategy that AML and other efficient matching systems employ to circumvent this problem is to store a sparse matrix with only the meaningful similarity scores (i.e., those above a certain threshold, such as 0.5). In the case of AML, this matrix is stored in the form of both a list of mapping candidates, to enable sorting and selection, and a *HashMap*-based table, to enable efficient searching. Each of AML’s matchers produces one such sparse matrix, or preliminary alignment, which can be combined with others either by simple union (keeping the highest score for the same mapping) or hierarchically (by adding only mappings from a less precise matcher that don’t conflict with those of more precise matchers).

### Handling the rich vocabulary of biomedical ontologies

#### Processing lexical annotations

AML, like most ontology matching systems that perform well in the biomedical domain, takes into account a wide range of lexical annotations from biomedical ontologies. Namely, AML stores in the *Lexicon* the local names (when not alphanumeric codes), labels, and all annotations with properties corresponding to labels or synonyms (e.g., “prefLabel”, “hasExactSynonym”, “FULLSYN”). The various annotations are condensed into four lexical categories: ‘localName’, ‘label’, ‘exactSynonym’, and ‘otherSynonym’. While this mapping is automatic, it covers the large majority of the annotation properties presently in use in biomedical ontologies and thesauri.

One strategy that, to the best of our knowledge, solely AML employs is that it assigns different numeric weights to each of its lexical categories, and uses these weights to score each mapping of lexical origin. The weighting scheme employed by AML is fixed, meaning that each lexical category is given a predetermined weight that reflect its expected reliability. This approach helps improve the effectiveness of AML’s *Selector* as it leads to less similarity ties and to mappings based on more reliable annotations being scored higher than those based on less reliable ones.

#### Inferring new synonyms

AML employs several strategies for automatically generating new synonyms, with the goal of improving the coverage and effectiveness of its hash-based searching algorithms. Having more synonyms increases the likelihood that corresponding concepts are described using equal lexical entries, and thus will tend to increase recall, but may also decrease precision.

One strategy AML employs is to automatically generate synonyms for classes by removing stop words from their names, using a predefined stop word list, as well as by removing name portions within parentheses. For example, for the SNOMED lexical entry “structure of nervous system”, AML generates the synonym “nervous system” by removing the leading stop words “structure” and “of”, and adds this synonym to *Lexicon* assigned to all classes for which the original entry was assigned. Analogously, for the NCI lexical entry “mixed mesodermal (mullerian) tumor”, AML generates the synonym “mixed mesodermal tumor” by removing the section within parentheses.

Another strategy AML employs for synonym generation consists in generating a thesaurus by comparing the various annotations of each class, and then using this thesaurus to generate new synonyms [12, 18]. For example, given a lexical analysis of the annotations ’stomach serosa’ and ’gastric serosa’ for Mouse Gross Anatomy Ontology (MA) class MA_0001626, AML would add to its thesaurus that ’stomach’ and ’gastric’ are synonymous words. It would then use this information to generate new synonyms for lexical entries containing either of the words by replacing it with the other. In order to contain the loss in precision that this strategy tends to generate, AML employs it in a dedicated matching algorithm, the *ThesaurusMatcher*, which finds only exact matches involving synonyms generated by the thesaurus.

Finally, AML can also use background knowledge sources to generate synonyms, but this strategy is detailed in the next subsection.

### Exploiting background knowledge

#### Background knowledge selection

The problem of automatically identifying relevant sources of background knowledge has been the subject of several studies [8, 9]. Most rely on analyzing the background knowledge sources to determine their overlap with the input ontologies, yet overlap does not imply usefulness. A background knowledge source is only useful if it contains (lexical or structural) knowledge not contained in the input ontologies and which is relevant to match them, or in other words, if we can find new mappings by using it (assuming it is reliable, and thus the mappings will mostly be correct). Given that, when employing a hash-based search algorithm, the difference in cost between computing a background knowledge alignment and computing an overlap is negligible, we might as well do the former and obtain a more direct measure of usefulness.

These are the foundations of AML’s algorithm for automatic selection of background knowledge sources [8]. This algorithm employs the concept of mapping gain, defined as the relative number of new mappings that an alignment would add to another alignment, as measure of usefulness. In a first stage, it uses the mapping gain over the baseline *LexicalMatcher* alignment to measure the individual usefulness of each candidate background knowledge source, and preselect them. In a second stage, it iterates through the preselected sources in descending order of individual mapping gain, recomputes the mapping gain over the current baseline alignment, and if significant, adds that background knowledge alignment to the baseline. Thus, it can not only identify the most promising individual background knowledge source, but also select a near-optimal combination of multiple background knowledge sources.

#### Information sources

Like most matching systems, AML relies primarily on the lexical information of background knowledge ontologies (*MediatingMatcher*). However, when OBO cross-references are available, it can use them instead of or in addition to the lexical information via its *XRefMatcher* [26]. Cross-references are essentially manually-curated mappings between an OBO ontology and others, listed in the ontology itself. For example, the UBERON class UBERON_0001275 (“pubis”) includes cross-references (via annotation property “hasDbXRef”) to FMA class 16595 (“pubis”) and NCI class C33423 (“pubic bone”). AML’s *XRefMatcher* employs these cross-references instead of performing lexical matches between the input ontologies and the background knowledge ontology, then like the *MediatingMatcher*, intersects the background knowledge alignments to derive an alignment between the two input ontologies. In the example above, if we were matching FMA to NCI using UBERON as a background knowledge source, it would map the FMA class to the NCI class because they are referenced by the same UBERON class.

Cross-references do not necessarily correspond to equivalence relations; all that is implied is a close semantic overlap. However, the same could also be said of ontology mappings: even if formally equivalence is always implied, the strictness with which it is meant varies from mapping to mapping. Thus, we found cross-references to be more reliable than literal lexical matches for inferring background knowledge mappings. For this reason, AML’s *XRefMatcher* supersedes its *MediatingMatcher*, as it uses cross-references when these are available, but complements them with lexical matches when the latter provide at least twice the coverage of the input ontology. Thus, it contemplates cases such as cross-references only being available for one of the input ontologies, as well as being available for both but only covering part of them.

#### Background knowledge usage

In addition to the traditional use of background knowledge ontologies as mediators, AML can also use them for lexical expansion, i.e., to generate new synonyms in the input ontologies. This strategy consists in adding, for each class of each of the ontologies to match that has a correspondence to a class of the background knowledge ontology, all the lexical entries of the latter as new synonyms. These correspondences must first be established by mapping the input ontologies to the background knowledge ontology, via either the *MediatingMatcher* or the *XRefMatcher*.

Given that the problem of handling large ontologies is compounded when using background knowledge ontologies, as not one but three matching tasks are required, the lexical expansion strategy enables AML to harness the knowledge contained in background knowledge ontologies more efficiently. It makes no difference from the use of background knowledge ontologies as mediators with regard to finding full-name matches, but it allows for partial matches to be indirectly derived from the background knowledge ontology with a single use (rather than three) of either the *WordMatcher* or the *StringMatcher*. However, deriving indirect partial matches can lead to a significant decrease in precision, meaning that this strategy can be less reliable than the mediating strategy.

### Using logical definitions

**Fig. 2:**
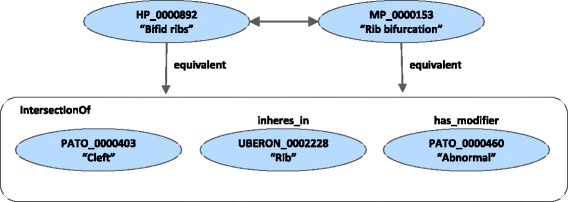
Example of a logical definition shared by a Human Phenotype Ontology and a Mammalian Phenotype Ontology class, which enables their mapping

AML has recently begun exploring the use of the logical definitions encoded in OBO Foundry ontologies [10] for ontology matching [12]. Logical definitions (or cross-products) correspond to composite mappings, where a class of one ontology is declared as equivalent to the intersection of two or more other classes of different ontologies. For example, the Human Phenotype Ontology (HP) [30] class HP_0000892 (“bifid ribs”) corresponds to Phenotypic Quality Ontology [31] class PATO_0000403 (“cleft”) inhering in the UBERON class UBERON_0002228 (“rib”) with modifier PATO_0000460 (“abnormal”), as depicted in Fig. 2. They are not strictly background knowledge in the sense that they are included in the ontologies themselves, but they do correspond to mappings to external ontologies. AML’s *LogicalDefMatcher* maps classes that have identical logical definitions. Continuing from the previous example, it would detect that Mammalian Phenotype Ontology (MP) [32] class MP_0000153 (“rib bifurcation”) has the exact same logical definition as HP_0000892 and thus map the two classes, as shown in Fig. 2. This is an example of a mapping that could not be found through lexical or structural matching approaches, but which logical definitions enable us to find.

### Evaluation

#### Datasets

The datasets used in this study were the OAEI 2016 datasets from the *Anatomy*, *Large Biomedical Ontologies*, and *Disease and Phenotype* tracks [14]:

- The *Anatomy* track consists of matching the Mouse Gross Anatomy Ontology [33] with the portion of the NCI Thesaurus [4] describing the human anatomy. It is evaluated using a manually curated reference alignment.
- The *Large Biomedical Ontologies* track features six matching tasks that consist in the pairwise matching of FMA [3], NCI [4], and SNOMED [5] in two modalities: small overlapping fragments, and whole ontologies. The evaluation is based on reference alignments derived automatically from the UMLS Metathesaurus [17].
- The *Disease and Phenotype* track includes two tasks, one consisting in mapping the Human Disease Ontology (DOID) [34] to the Orphanet and Rare Diseases Ontology (ORDO), and another consisting of mapping the Human Phenotype Ontology (HP) [30] to the Mammalian Phenotype Ontology (MP) [32]. The evaluation carried out in the OAEI 2016 was primarily based on consensus alignments that include all mappings found by either 2 or 3 participating matching systems.

#### Settings

To evaluate the impact of the various challenges of matching biomedical ontologies and the strategies for tackling them, we conducted a number of tests, which are further detailed in the “Results” section.

All tests were carried out in a personal computer with an Intel i5-4570 CPU @ 3.20GHz, with 10GB RAM allocated to Java, and Windows 7 64-bit operating system. Except were otherwise noted, the *StringMatcher* was run concurrently on 4 CPU threads, and all other matching algorithms were run using a single CPU thread.

When AML’s complete matching pipeline is mentioned, it refers to the matching pipeline employed for the OAEI 2016 [12]. The sources of background knowledge available to AML were also the same as it used in the OAEI 2016: the Uber Anatomy Ontology (UBERON) [2], the Human Disease Ontology (DOID) [34], and the Medical Subject Headings (MeSH) [35].

Tests where only the run time was being assessed were carried out in all datasets. Tests where the F-measure was being assessed were carried out in only the *Anatomy* and *Large Biomedical Ontologies* datasets (except where otherwise noted) since a consensus alignment, as used in the evaluation of the *Disease and Phenotype* track, was deemed insufficiently accurate for the purpose of this study.

In the final test of this study, we performed a manual evaluation of the mappings found uniquely through logical definitions from the HP-MP task (as logical definitions are only available for the ontologies in this task). These mappings were produced with older versions of the logical definitions of the HP ontology, which mapped to the FMA rather than to UBERON. Thus to derive HP-MP mappings based on logical definitions, the cross-references between UBERON and FMA were used to provide correspondences between the logical definitions, when the definitions were otherwise identical.

## Results

### Efficiency tests

#### Hash-based searching versus pairwise comparisons

In order to compare the efficiency of hash-based searching with traditional pairwise comparison algorithms, we implemented a functional equivalent of AML’s *LexicalMatcher* that makes pairwise equality comparisons instead of hash-based searches. We compared the run time of this *QuadraticLexicalMatcher* (running concurrently on 4 CPU threads) with that of the *LexicalMatcher*. Furthermore, we performed a power law regression of the run times of the two approaches as function of the number of lexical entries in the matching task.

**Fig. 3:**
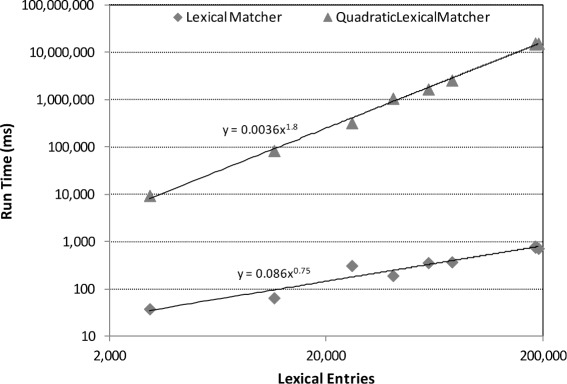
Run time versus number of lexical entries, in a log-log scale, for the hash-based searching *LexicalMatcher* and its functional equivalent but pairwise comparing *QuadraticLexicalMatcher*. Power law regression curve and equation are shown for each algorithm. The *LexicalMatcher* was executed using a single CPU thread, whereas the *QuadraticLexicalMatcher* ran asynchronously on 4 CPU threads

The results of this comparison are shown graphically in Fig. 3, and detailed in Table 2. The difference in scale between the run times of the two approaches is readily apparent, as the *LexicalMatcher* runs in under a second for even the largest tasks whereas the *QuadraticLexicalMatcher* ranges from 9 seconds for the *Anatomy* task to over 4 hours for the three whole ontologies tasks of the *Large Biomedical Ontologies*. The power law regressions reveal that the *LexicalMatcher* has a sub-linear behavior (exponent 0.75) as function of the number of lexical entries, whereas the *QuadraticLexicalMatcher* has a near-quadratic behavior (exponent 1.8).

**Table 2:** Run time comparison between the hash-based *LexicalMatcher* (in milliseconds) and its functional equivalent *QuadraticLexicalMatcher* that performs pairwise comparisons (in seconds), on all biomedical OAEI 2016 tasks

Task	*LexicalMatcher*		*QuadraticLexicalMatcher*	
	Hash-based searches	Time (ms)	Pairwise comparisons	Time (s)
Anatomy	3,072	38	15,587,328	9
FMA-NCI small	11,515	65	224,715,225	84
FMA-SNOMED small	26,295	311	959,057,535	323
DOID-ORDO	40,683	191	2,892,154,470	1,071
HP-MP	59,240	358	3,696,339,040	1,672
SNOMED-NCI small	76,134	372	5,803,999,356	2,579
FMA-SNOMED whole	184,484	761	41,136,795,772	14,984
SNOMED-NCI whole	184,484	801	35,189,031,612	15,640
FMA-NCI whole	190,743	721	42,532,446,369	15,509

#### Local versus global string matching

The application of traditional string matching algorithms, such as ISub [27], requires pairwise comparisons and thus is not scalable. Thus many matching systems forgo their use, instead opting for approximations based on hash searches (such as n-gram overlap). AML is able to make use of string matching algorithms by employing them locally, in the vicinity of mappings derived through hash-based searching. The expectation is that this local matching strategy scale approximately linearly with the size of the ontologies (in number of classes rather than lexical entries, as it is at its core a structural algorithm). To assess whether that is the case, we measured the run time of AML’s *StringMatcher* when used (locally) in its full matching pipeline and performed a power law regression as function of the number of classes (of the input ontology with the most classes).

**Fig. 4:**
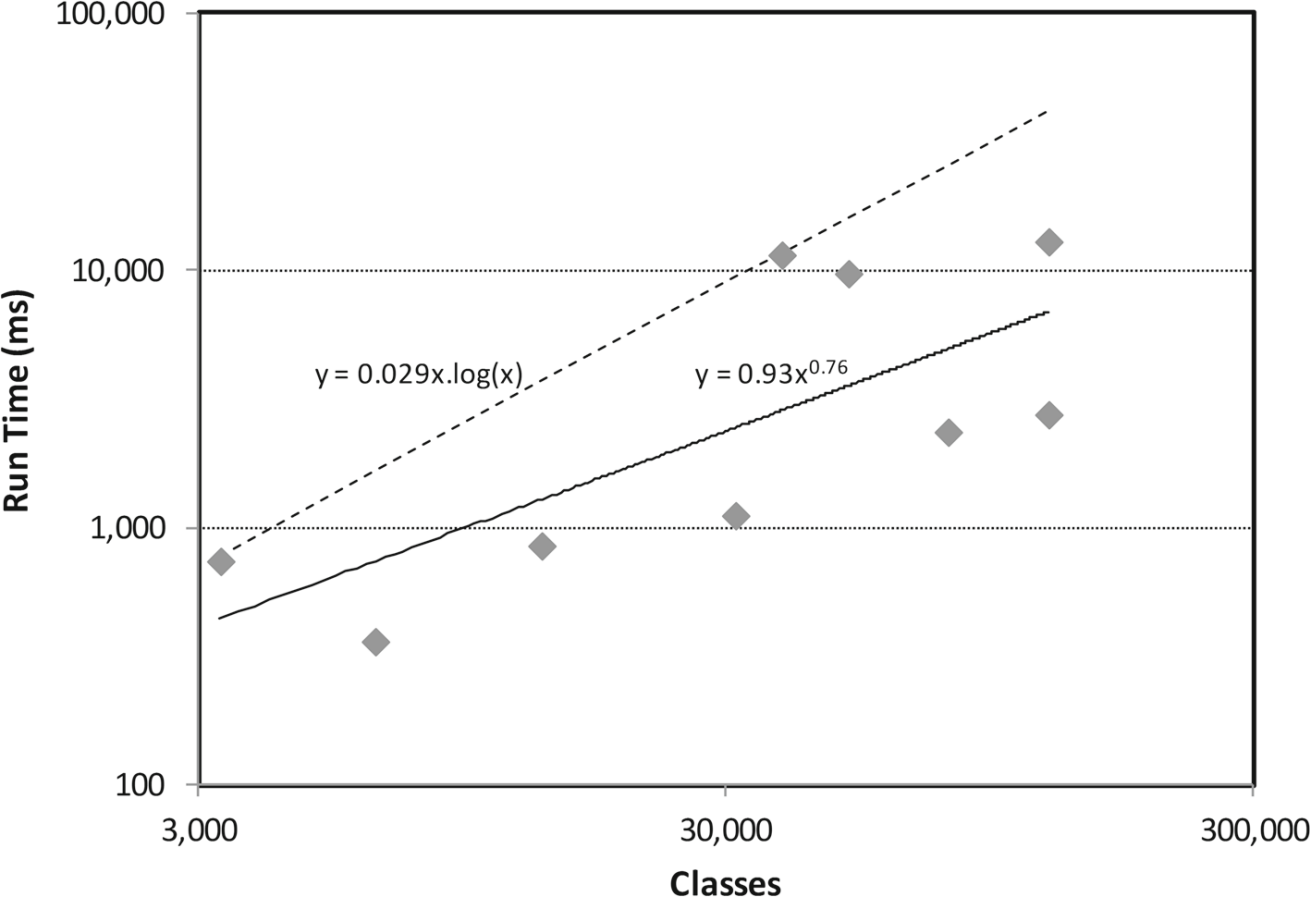
Run time versus number of classes, in a log-log scale, for the *StringMatcher* applied locally, in the neighborhood of the mappings found by the other matching algorithms in AML’s pipeline. Both power law regression curve and equation, and bounding n.log(n) curve and equation are shown. The algorithm ran asynchronously on 4 CPU threads

The results of this regression, shown in Fig. 4, reveal that the behavior of the local *StringMatcher* is on average sub-linear (exponent 0.76), and while there is substantial variation from this behavior, it is bound by *O*(*n* log(*n*)). In more concrete terms, the local *StringMatcher* runs in 13 seconds in the worst case, whereas the global algorithm has an expected run time of over 8 hours for the three whole ontologies tasks of the Large Biomedical Ontologies.

To assess the effectiveness of applying string matching only locally in comparison with applying it globally, we ran AML’s full matching pipeline replacing the local *StringMatcher* with a global run of that algorithm, and compared the F-measure of the pipeline with the global and local variants. We did not run this test on the *Large Biomedical Ontologies* whole ontologies tasks, as the expected run time of the global *StringMatcher* in these tasks exceeds 8 hours, and the conclusions drawn from the small overlapping fragments tasks can be extrapolated to these tasks. The results of this comparison are shown in Table 3.

**Table 3:** Evaluation of AML’s full matching pipeline with the *StringMatcher* run locally and run globally

Task	Local *StringMatcher*				Global *StringMatcher*			
	Prc	Rec	F-m	Time (s)	Prc	Rec	F-m	Time (s)
Anatomy	95.0%	93.6%	94.3%	0.74	94.1%	93.5%	93.8%	141
FMA-NCI small	95.8%	91.0%	93.3%	0.36	95.1%	91.5%	93.2%	164
FMA-SNOMED small	92.3%	76.2%	83.5%	0.85	88.2%	79.7%	83.8%	796
SNOMED-NCI small	91.4%	73.6%	81.6%	9.7	86.1%	75.5%	80.5%	5010

Precision (Prc), Recall (Rec), and F-measure (F-m) of the alignment produced by each strategy, and execution time of the *StringMatcher* algorithm

The results show that, as expected, employing the global *StringMatcher* has an advantage with respect to recall in most tasks (the exception being *Anatomy*). The counterpoint is that precision is significantly lower than when the local variant is employed, to the effect that the F-measure is also lower in most tasks (except for *FMA-SNOMED small*).

### Lexical richness tests

#### All lexical annotations versus primary annotation

The number and variety of lexical annotations per class are a feature of biomedical ontologies that should be taken into account when matching them. In order to assess the impact of this lexical richness, we compared the performance of AML’s *LexicalMatcher* when using all available lexical annotations (as normal) and when using only the primary name of each class. To avoid introducing an external bias, we turned off AML’s automatic generation of synonyms for this test, so that only the lexical richness of the ontologies themselves is considered.

The results of this test, as shown in Table 4, are conclusive in that the effect of considering all lexical annotations leads to a substantial increase in F-measure in all tasks, ranging from 4.7% in the case of *Anatomy* to 18.3% in the case of the *FMA-NCI small* task. They demonstrate that taking into account all lexical annotations of biomedical ontologies is clearly necessary to match them effectively.

**Table 4:** Comparison between the *LexicalMatcher* using all class names and synonyms, and using only primary names

Task	All names & synonyms			Primary name		
	Precision	Recall	F-measure	Precision	Recall	F-measure
Anatomy	96.1%	69.7%	80.8%	99.7%	61.5%	76.1%
FMA-NCI small	97.5%	77.5%	86.4%	99.5%	51.7%	68.1%
FMA-SNOMED small	98.8%	19.9%	33.2%	99.2%	15.1%	26.2%
SNOMED-NCI small	96.5%	52.6%	68.1%	98.9%	40.6%	57.6%
FMA-NCI whole	69.5%	77.5%	73.3%	96.7%	51.7%	67.4%
FMA-SNOMED whole	94.6%	19.9%	32.9%	96.6%	15.1%	26.1%
SNOMED-NCI whole	86.7%	52.6%	65.5%	96.8%	40.6%	57.2%

#### Weighted versus unweighted lexical annotations

To evaluate the contribution of differentiating between different kinds of lexical annotations, we ran AML’s full matching pipeline with its weighting scheme turned off, and compared the results to those of the normal pipeline. The results of this comparison, shown in Table 5, reveal that the use of *Lexicon* weights improves the F-measure in all matching tasks except for *FMA-SNOMED small* where there is a tie. The most extreme case is that of the *Anatomy* task, where the F-measure increases by 6.3%.

**Table 5:** Comparison between AML’s full matching pipeline with and without the use of *Lexicon* weights to score the mappings

Task	*Lexicon* weights			No *Lexicon* weights		
	Precision	Recall	F-measure	Precision	Recall	F-measure
Anatomy	95.0%	93.6%	94.3%	81.3%	95.8%	88.0%
FMA-NCI small	95.8%	91.0%	93.3%	94.6%	91.1%	92.8%
FMA-SNOMED small	92.3%	76.2%	83.5%	90.9%	77.3%	83.5%
SNOMED-NCI small	91.4%	73.6%	81.6%	89.9%	74.1%	81.2%
FMA-NCI whole	83.8%	87.2%	85.5%	78.3%	86.4%	82.2%
FMA-SNOMED whole	88.0%	69.0%	77.4%	84.4%	70.3%	76.7%
SNOMED-NCI whole	89.7%	67.1%	76.8%	86.4%	67.1%	75.6%

#### The contribution of the *ThesaurusMatcher*

AML’s *ThesaurusMatcher* exploits the lexical richness of biomedical ontologies to infer new synonyms through automatic lexical composition analysis, and thereby find new mappings. In order to assess the extent to which new knowledge can be generated by such an approach, and how reliable it is, we compared the performance of *LexicalMatcher* plus *ThesaurusMatcher* with the performance of the *LexicalMatcher* alone. The results of this comparison are presented in Table 6.

**Table 6:** Comparison between the combination of *LexicalMatcher* and *ThesaurusMatcher*, and the *LexicalMatcher* alone

Task	Lexical+Thesaurus			Lexical only		
	Precision	Recall	F-measure	Precision	Recall	F-measure
Anatomy	95.7%	71.4%	81.8%	96.1%	69.7%	80.8%
FMA-NCI small	95.8%	83.7%	89.3%	96.8%	81.8%	88.7%
FMA-SNOMED small	96.9%	62.9%	76.3%	97.8%	62.2%	76.0%
SNOMED-NCI small	95.5%	59.9%	73.6%	95.9%	59.1%	73.1%
FMA-NCI whole	63.0%	82.9%	71.6%	65.4%	81.8%	72.7%
FMA-SNOMED whole	90.1%	62.8%	74.0%	92.8%	62.2%	74.5%
SNOMED-NCI whole	81.3%	59.7%	68.8%	82.0%	59.1%	68.7%

We can see that the *ThesaurusMatcher* leads to a consistent increase in recall, but decrease in precision in all tasks. For *Anatomy*, and the *Large Biomedical Ontologies* small tasks the balance is positive, as the resulting F-measure is greater than without the *ThesaurusMatcher*. For *FMA-NCI whole* and *FMA-SNOMED whole*, it is negative, whereas for *SNOMED-NCI whole*, it is essentially neutral.

### Background knowledge tests

#### Comparison of information sources and usage strategies

There are two main strategies for using background knowledge ontologies: as mediators, or for lexical expansion. There are also two types of information that can be used to map the background knowledge ontologies to the input ontologies: lexical information, and cross-references. We evaluated AML’s full matching pipeline with the background knowledge matching component modified appropriately to cover all four combinations of these two factors. We carried out this evaluation on the *Anatomy* and *FMA-NCI small* tasks, as these are the only tasks in which the coverage of the available cross-references from UBERON is comparable to its lexical coverage, and thus for which comparing the two information sources would be fair. The results of this evaluation are shown in Table 7.

**Table 7:** Evaluation of AML’s matching pipeline in the Anatomy and FMA-NCI small tasks with different combinations of background knowledge information source (lexical vs. cross-references) and usage strategies (mediator vs. lexical expansion)

Task	BK info	BK usage	Precision	Recall	F-measure
Anatomy	Lexical	Mediator	94.8%	91.0%	92.9%
	(1365-1352)	Expansion	94.6%	90.2%	92.4%
	Cross-refs	Mediator	93.4%	92.5%	93.0%
	(1389-1401)	Expansion	95.0%	93.6%	94.3%
FMA-NCI small	Lexical	Mediator	93.9%	91.7%	92.8%
	(1624-1598)	Expansion	93.6%	91.5%	92.5%
	Cross-refs	Mediator	95.8%	91.0%	93.3%
	(1541-1559)	Expansion	94.2%	92.0%	93.1%

The number of classes of the two input ontologies covered by each information source is shown within parentheses below the source in each dataset

The first observation we can make from the results is that cross-references are the best source of information in both tasks, albeit with different usage strategies, and are better than using lexical information regardless of strategy. The lexical expansion strategy produces strictly worse results than the mediator strategy when based on lexical information. When based on cross-references, it produces a higher recall than the mediator strategy, and in the case of the *Anatomy* task, a higher F-measure as well.

#### On the use of logical definitions

Another source of information that can be exploited for matching biomedical ontologies are OBO logical definitions. AML and PhenomeNET [21] both explored the use of logical definitions in the OAEI 2016’s HP-MP task from the *Disease and Phenotype* track.

Because there is no manually validated reference alignment for the HP-MP task, we assessed the contribution of the *LogicalDefMatcher* by manually evaluating the mappings found by this matcher and not by AML’s pipeline when this matcher is disabled. We classified each mapping as: *equivalent*, if the two classes were deemed semantically equivalent; *overlapping* if the classes were not strictly equivalent (one was slightly broader than the other) but sufficiently similar that a direct mapping between the two would be conceivable depending on the scope of the alignment; or *false*, if the classes were too dissimilar to be mapped. The full results of this manual evaluation are included in the Additional file 1.

Out of the 92 mappings identified only with the *LogicalDefMatcher*, we found that 49 were equivalent, and an additional 21 were overlapping, therefore in total 70 mappings were plausibly correct. This gives us a best case precision of 76.1%, and a worst-case precision of 53.3% (if we consider only the strictly equivalent mappings correct). Given that these 92 mappings represent 5% of the total mappings found by AML, their contribution to AML’s recall should be significant even in the worst case, but in the absence of a reference alignment, we cannot determine it, nor can we ascertain whether the contribution of this matcher is positive with respect to F-measure.

## Discussion

### Efficiency tests

The evaluation of the hash-based searching strategy against the traditional pairwise comparison strategy leaves no room for doubt that hash-based searching is scalable, and able to tackle even the largest ontology matching problems efficiently. One aspect that our test did not contemplate (as it would be impossible to do so without developing an ontology loading algorithm anew) is that building the *HashMap* data structures required for hash-based searching has a cost in both run time and memory in comparison to using simpler data structures such as lists. However, this cost is, in practice, negligible. With respect to run time, populating a *HashMap* has *O*(n) complexity (even if it is slower than populating a list) and thus is largely compensated by the reduction in the complexity of the matching process, even for small ontologies. *HashMaps* do require significantly more memory than lists, but even for the largest ontologies, the total memory requirements are unremarkable for modern computer standards. For instance, AML’s total memory requirement for loading the largest ontologies in the OAEI is under 1.8 GB, of which 60% are spent by the OWL API alone, and the whole loading process takes approximately 1 minute in the machine used in testing (of which under 25% is spent on populating the *LexiconHashMaps*). Thus, it is clear that hash-based search strategies should replace traditional pairwise comparisons whenever possible, be it for finding equal lexical entries, or entries with overlapping words or n-grams.

While computing traditional string similarity requires pairwise matching, our evaluation shows that AML’s local string matching strategy is an effective search space reduction strategy. It enables the matching of even the largest ontologies in seconds, whereas performing a full global string match would take several hours. Moreover, the results suggest that the strategy is scalable, with a worst case *O*(*n* log(*n*)) complexity with respect to the size of the ontologies.

In addition to being scalable, a search space reduction strategy should at least approximate the results of performing a full space search in order to be useful. Our experiments revealed that, as expected, there is some loss of information when performing a local string match, as the recall of the global string match is usually higher (with the exception of the *Anatomy* task). However, the local string match has an advantage in terms of precision, which more than compensates for its lower recall, leading to a higher F-measure in most tasks (except *FMA-SNOMED small*). Thus, the results indicate that the local string matching strategy is actually more effective than global string matching, in addition to being much more efficient.

The reason for this advantage in effectiveness is tied with the issue of the lexical complexity of biomedical ontologies. Although there are many similar lexical variants of the same concept that can be found through a string similarity metric, there are also many cases of different concepts that have similar names, which will also be matched with such a metric (e.g., ‘olfactory receptor nerve’ and ‘olfactory receptor neuron’). By matching only classes in the vicinity of previously found (usually high-quality) mapping candidates, we are able to exclude many of these false positives, with some loss of true positives, but resulting in a better alignment overall.

Evidently, one factor behind the effectiveness of the local string matching strategy is that the preliminary alignment prior to its execution was already fairly extensive, thanks to the use of adequate background knowledge as well as the *WordMatcher*. However, our goal was precisely to assess whether a complete matching system such as AML, which relies solely on hash-based searching or otherwise scalable matching techniques, would stand to improve by employing traditional (global) string matching, were efficiency considerations put aside. The results clearly point to the conclusion that it wouldn’t.

The local approach to string matching is, in a sense, a fusion between a structural and a lexical matching algorithm, and it combines advantages of the two approaches. One the one hand, pure structural matching algorithms are often unreliable for biomedical ontologies due to their modeling differences, so making string similarity comparisons to validate potential structural matches makes sense. On the other hand, as we have shown, string matching can generate many false positives in the biomedical domain, so it also makes sense to filter string matches using structural information. Evidently, if the conclusion is that we want to filter string matches with structural information, then it is vastly more efficient to consider that information a priori, as the difference in run time between the local and global *StringMatcher* makes clear.

### Lexical richness tests

Our lexical richness experiments have made clear that the number and variety of lexical annotations per class are a feature of biomedical ontologies that should be taken into account when matching them.

With respect to number, the results were conclusive in showing that considering all lexical annotations available, as most state-of-the-art systems do, is more effective than considering only the primary name of each class. The rich biomedical vocabulary is filled with synonyms, with their usage varying between communities, and only by making use of all available synonyms can biomedical ontologies from different communities be effectively bridged.

Concerning variety, the results showed that taking into account the different levels of precision of the various types of synonyms by weighting is clearly advantageous over considering them all on equal footing. This is unsurprising given that broad and narrow synonyms are, by definition, less precise than exact synonyms or labels. Nevertheless, these results do not imply that it is necessary to explicitly take into account the reliability of lexical annotations. After all, the experiment is inextricably tied to AML’s filtering strategy, and it is plausible that with a different strategy (e.g., one that made use of structural information) such a necessity would not be felt. Moreover, strategies other than weighting could be devised for taking the reliability of lexical annotations into account. That said, AML’s weighting strategy is likely the simplest strategy for doing so, and its extremely low computational cost leads us to posit that other matching systems would stand to gain by adopting a similar strategy, provided that the weighting scheme employed reflects the precision of the different synonyms and the matching thresholds are revised to take the weighting into account.

Our experiment with AML’s *ThesaurusMatcher* showed that, while it is essential to account for the lexical complexity of biomedical ontologies when matching them, that complexity can also be exploited to generate new knowledge. More concretely, lexical composition analysis can be applied automatically to generate new synonyms, which can then be used to help match the ontologies. The fact that, at present, this strategy is unreliable for very large ontologies suggests that the algorithm may not be sufficiently mature, but we expect that with further development it can be refined to address these limitations.

There are a few oddities in the results that merit further comment. The extremely low recall observed in the *FMA-SNOMED* tasks in the first test (Table 4) is a consequence of the peculiar terminology SNOMED employs for anatomical structures. The names of most of these structures actually feature the word “structure” (e.g., “structure of nervous system”) whereas FMA simply lists the name of the structure itself (e.g., “nervous system”). AML handles this variation through its stop-word synonym generator, but when it is turned off, as it was in this test, finding these matches becomes impossible using a literal lexical matcher alone (as used in the test). The low precision in the *FMA-NCI whole* task in the first test (Table 4), when using all names and synonyms, is due to the fact that the NCI includes both a branch devoted to human anatomy and a branch devoted to mouse anatomy, and only the former is mapped to FMA in the reference alignment (because it is derived automatically from UMLS). The NCI mouse anatomy classes all include the acronym “MMHCC” at the end of their primary names, and for this reason are not matched when only primary names are used, but they do include synonyms without this acronym which are identical to human anatomy names, and thus are matched when all lexical annotations are considered. The extreme loss in precision in the *Anatomy* task in the second test (Table 5), when no weights are used, is due to the fact that this is the only task in which lexical expansion is (automatically) used by AML, which leads to an increase in the number of conflicting mappings. Without its weighting system, AML cannot effectively filter the alignment, which leads to a worse precision.

### Background knowledge tests

Exploiting background knowledge effectively is a key factor to successfully matching biomedical ontologies, as evidenced by the results of the OAEI’s biomedical tracks [14]. Two key aspects in using background knowledge ontologies are what information source to use to map them with the input ontologies, and how to make use of them once they are mapped.

With respect to the information source, our experiments indicated that cross-references perform better than lexical information. This, of course, is conditional on cross-references being available (which is presently only true for some OBO ontologies) and their coverage being extensive. Even when they are not extensive, we can generally expect cross-references to be more reliable than lexical information, with the caveat that the intended scope of the alignment agree with that of the cross-references. That may not always be the case: for example, as we mentioned previously, the NCI ontology includes a branch on mouse anatomy (not present in the small task) that in the UMLS-based reference alignment is not mapped to the FMA (since the UMLS is primarily focused on human health) but in the UBERON cross-references is (as UBERON is a multi-species anatomy ontology). Were we to tackle the *FMA-NCI whole* task, we might be able to exclude the mouse anatomy branch with lexical information (thanks to the presence of the “MMHCC” acronym) but we wouldn’t be able to do so with the UBERON cross-references. Thus, if the intended scope of our alignment were human health, we would likely be better off using lexical information, but if it were broader, the UBERON cross-references would be best.

Concerning the usage strategy, our results were not fully conclusive as to whether mediator usage or lexical expansion is the better strategy. However, there are some discernible patterns that merit discussion. When the information source is lexical, it is clear that lexical expansion leads to strictly worse results than mediator usage. This may seem surprising, as the reasoning behind the lexical expansion strategy is to enable word and string matching to be applied using the information from the background knowledge source. Thus, we would expect that lexical expansion lead to at least a higher recall. The catch is that in performing expansion, the indirect matches from the background knowledge source are put on par with the direct lexical matches between the input ontologies, and this apparently leads to cases of wrong mappings being selected instead of correct ones. When cross-references are the information source, lexical expansion does lead to an increase in recall over mediator usage in both datasets, which shows that the reasoning behind the lexical expansion strategy is not without merit.

The observation that, with cross-references, lexical expansion leads to an increase in F-measure as well for *Anatomy* but a decrease for *FMA-NCI small* brings us to murky territory, as it is tied to the fact that even among human experts there is disagreement on what constitutes a mapping. Consider that both the *Anatomy* reference alignment and the UBERON cross-references between these ontologies are manually curated and have as scope inter-species anatomy correspondences. Yet, the *Anatomy* reference alignment is more extensive (1516 vs. 1409 mappings) and their agreement is high but only partial (1342 mappings in common). Debating whether one is more correct than the other goes beyond the scope of this manuscript. What we can say, is that by happenstance, employing the lexical expansion strategy with UBERON cross-references brings the resulting alignment closer to the *Anatomy* reference alignment. In the *FMA-NCI small* task things are different in that the extension of the reference alignment is substantially greater than that of the UBERON cross-references (2686 vs. 1460 mappings) because they differ in scope (both the FMA and NCI include a genetic component that is not present in UBERON, but is present in the UMLS-derived reference). In this case, performing lexical expansion does enable the finding of more correct mappings, but at the cost of a greater loss in precision and consequent loss in F-measure.

OBO logical definitions, like cross-references, represent an additional, non-lexical source of information that can be used for ontology matching. Whereas cross-references are direct mappings between an ontology and another, logical definitions are composite mappings between an ontology class and two or more classes from different external ontologies with the intent of defining the former class semantically (as depicted in Fig. 2, and described in the “Methods” section). Our evaluation has made clear that logical definitions are a less reliable source of information for matching than cross-references, undoubtedly due to their composite nature, which means that using logical definitions in an automated ontology matching scenario is risky. However, given that they are able to find mappings that are not identifiable through lexical methods, using them in a semi-automated scenario, where users are available for validation, would be promising.

## Conclusions

Biomedical ontologies pose several challenges to ontology matching due to their size, their lexical richness and complexity, their often profound modeling differences, and the necessity of using background knowledge to match them effectively. In this study, we dissected and evaluated several strategies for tackling these challenges, using the AML matching system as a platform.

Our efficiency tests have conclusively demonstrated the scalability of the hash-based searching strategy for ontology matching, in contrast with the traditional pairwise comparison strategy. Furthermore, they showed that AML’s local string matching strategy is not only scalable but also effective, as it produces more accurate results than performing global pairwise string matching. We foresee that much of the future development in ontology matching will be devoted to adapting traditional matching algorithms to the hash-based searching paradigm, or devising search space reduction strategies, such as local matching, in order to make them more scalable.

Our lexical richness tests were also conclusive in showing that accounting for the various types of lexical annotations of biomedical ontologies is critical for matching them effectively, as the gain in recall far outweighs the loss in precision. Furthermore, they showed that accounting for the reliability of the different annotation types is relevant, and that a weighting scheme such as the one of AML is a sound strategy for doing so. Last, but not least, we showed that the lexical richness of biomedical ontologies can be exploited to derive additional lexical knowledge, and consequently find new mappings. However, AML’s algorithm for exploiting this information requires further refining and evaluation.

Our background knowledge tests showed that using background biomedical ontologies for lexical expansion is usually a less reliable strategy than using them simply as mediators, which validates the prevalence of the latter strategy in state-of-the-art ontology matching systems. The tests also revealed the value in exploiting the efforts of the OBO foundry to map ontologies, by means of both cross-references and logical definitions. Our conclusion that cross-references are more reliable than lexical information for mapping a background knowledge ontology to the input ontologies is hardly surprising, given that cross-references are essentially manually curated mappings. But while it suggests that cross-references should be used whenever available, care should be taken with respect to the overlap between the intended scope of the alignment and that of the cross-references. Logical definitions are an interesting source of information upon which to derive mappings, as they enable us to map classes based purely on their semantics, and capture mappings that could not be identified from syntax. However, the semantic latitude of the classes to which these logical definitions point means that some mappings derived from them would be expectedly wrong. We confirmed by manual evaluation that the precision of the mappings derived uniquely from logical definitions was not very high, which suggests that this approach is best suited for semi-automated ontology matching scenarios.

Although our evaluation relied on AML as a testing platform, we expect the conclusions drawn to be generally applicable to any matching system tackling biomedical ontologies. Some of the features tested are at present only implemented by AML, and thus replicating the tests in other matching systems would require substantial development. Nevertheless, AML’s matching algorithms are mostly equivalent to those of other state of the art systems, and we do not expect any of the configurations specific to AML to bias the factors tested (except for the weighting strategy in the lexical weighting test, evidently). Since all tests are comparative, basing them on a different matching system should lead to the same overall conclusion, even though the absolute results would be likely different.

Overall, our study made clear that the challenges posed by biomedical ontology matching are complex, and are still far from being fully addressed. They demand efficient matching systems that are able to combine multiple strategies into a mature matching approach.

Two challenges of matching biomedical ontologies that our study did not delve into are the impact of modeling differences on alignment repair and the relevance of “part of” relations. Alas, the OAEI benchmarks where repair is a substantial problem lack manually curated reference alignments, and atypically, the “part of” relation is poorly represented in the OAEI biomedical benchmarks, which impedes any serious evaluation of these two aspects. Another aspect that is not covered by our study or by the OAEI benchmarks is the semantics of the ontology mappings. Even though previous research has tackled the issue of inferring mappings with different semantics [36, 37], present OAEI benchmarks focus exclusively on finding equivalence mappings, and as a result so do most state of the art matching systems. Since the OAEI is one of the major driving forces behind ontology matching development, it is our view that more effort is needed to ensure that the biomedical tracks of the OAEI are adequate in both their coverage of pertinent ontology matching challenges, and in the quality of their evaluation. Simply adding new benchmarks is of little worth to the community unless these cover new relevant challenges and the quality of the evaluation enables the assessment of such challenges.

## Additional file

Additional file 1 Manual evaluation of the HP-MP mappings found through logical definitions Tab-separated text file listing each mapping (URI and label of the source and target classes) and its classification. (TSV 13 kb)
